# DNA Methylation Pattern of CALCA and CALCB in Extremely Premature Infants with Monochorionic Triplets after Single-Embryo Transfer

**DOI:** 10.1155/2021/1438837

**Published:** 2021-10-05

**Authors:** Feng Gao, Yujia Guo, Xingting Chen, Qiuyang Gu, Shirong Huang, Qingquan Chen, Xiaoming Xu, Kai Zeng, Huilin Zhou, Yilu Zou, Qicai Liu

**Affiliations:** ^1^Department of Pathology, 1st Affiliated Hospital, Fujian Medical University, Fuzhou 350004, China; ^2^Center for Reproductive Medicine, 1st Affiliated Hospital, Fujian Medical University, Fuzhou 350004, China; ^3^Department of Medical Ultrasonography, 1st Affiliated Hospital, Fujian Medical University, Fuzhou 350005, China; ^4^Department of Laboratory Medicine, Fujian Medical University, 350004 Fuzhou, China; ^5^Beijing Perfect Family Hospital, Beijing 100032, China; ^6^Department of Anesthesiology, 1st Affiliated Hospital, Fujian Medical University, Fuzhou 350005, China

## Abstract

Compared with full-term peers, premature infants are more likely to suffer from neonatal diseases and death. Variations in DNA methylation may affect these pathological processes. Calcitonin gene-related peptide (CGRP) plays a complex and diversified role in reproduction and chronic inflammation, and participates in the functional maintenance of vascular adaptation and trophoblast cells during pregnancy. Here, premature live births with single-chorionic triple embryos after single-embryo transfer were used as research objects, while full-term infants with double embryos and double-chorionic twins were used as controls. DNA was extracted from umbilical cord tissues for pyrosequencing to detect the methylation level of CpG island in CGRP promoter region. The average values of CGRP methylation in the umbilical cord tissues of very premature fetuses were higher than that of normal controls obtained from the databases. Immunofluorescence results showed that the expression of *α*CGRP was decreased in the blood vessel wall of the umbilical cord of monozygotic triplets, especially in death cases, while the *β*CGRP had a compensatory expression. In conclusion, our findings suggest that hypermethylation of CGRP might be considered as an important cause of serious neonatal morbidities.

## 1. Introduction

Impaired blood circulation in the umbilical cord or placenta might lead to intrauterine growth restriction and premature delivery and is considered as an important complication during pregnancy [1]. Calcitonin gene-related peptide (CGRP) is regarded as the most effective vasodilator, and it plays a complex and diversified role in chronic low-grade inflammation [2]. It is closely related to functional maintenance of vascular adaptation and trophoblast cells during pregnancy [3]. It comprises two subtypes CALCA (*α*CGRP) and CALCB (*β*CGRP), and these coordinate with molecular signaling network of decidualization, placenta formation, and fetal growth to promote successful pregnancy outcomes [3, 4]. But the role of it in finely synchronized molecular and cellular events during preterm labor still remains to be unknown.

Our previous studies have revealed that abnormal secretion of *β*CGRP is caused by CALCB mutation, which lose its inhibitory effect on inflammatory cells, resulting in obliterative vasculitis and perineuritis [5]. At the same time, interleukin- (IL-) 6, IL-10, and tumor necrosis factor-*α* (TNF-*α*) were highly expressed in CGRP-KO rats, which might be involved in the occurrence of fetal inflammation [6]. CGRP can inhibit the synthesis and/or release of inflammatory factors such as TNF-*α*, IL-1, and IL-12 by regulating cytokine expression of macrophages through cAMP/PKA signaling pathway and inhibiting the differentiation of Th1 lymphocytes. CGRP can also regulate the balance between Th1/Th2 cells, promoting the release of IL-10 and IL-4 from macrophages and Th2 lymphocytes and inhibiting the antigen presentation of Th1 cells, thereby inhibiting Th1 cell-mediated cellular immunity [7, 8]. Therefore, we speculated that CGRP might participate in immune regulation and inhibition of inflammation during pregnancy, thereby affecting fetal development and progression of labor.

In addition to the influence of neurological factors, CGRP expression also plays an important regulatory role in its own gene structure and methylation modification. There is a large CpG island in the 5′ flanking promoter region of the CALCA and contains a microbial infection-specific response elements that regulate the transcription of procalcitonin (PCT) during bacterial infection [9]. The structure of CALCB is similar to that of CALCA, and it consists of two different CpG-rich regions, wherein one is located around exon 1, and the other is located about 1.5 kb upstream [10]. Single-egg multiple births might occur due to different methylation patterns during embryonic development, and this might be a reason for the differences in fetal development [11]. The monochorionic triplet (MCTA) genetic background is a common, reliable, and excellent sample for studying the role of CGRP in pregnancy maintenance. Hence, in this study, the methylation levels and expression location of CALCA and CALCB in the umbilical cord tissues of monochorionic three amniotic sacs were detected, and the relationship between them and the very preterm birth were evaluated.

## 2. Materials and Methods

### 2.1. Research Subject

A case of monochorionic triplets and the very premature live births of three boys after single blastocyst transplantation were collected as research subjects from the Reproductive Medicine Center of the First Affiliated Hospital of Fujian Medical University in April 2020. In addition, those undergoing double blastocyst transplantation in the same period were selected. Chorionic twins and two full-term live births were used as controls. General information including age, basic hormone levels, fetal development process, and family history were collected. After obtaining their consent, the remaining umbilical cord and placental tissue samples from *in vitro* pathological diagnosis were collected for methylation and immunohistochemical testing.

### 2.2. Database Retrieval and Prediction

To study the relationship between gene methylation in umbilical cord or cord blood and preterm birth, two matching databases were found. Genome wide DNA methylation profiling of 152 umbilical cord blood samples from CCCEH birth cohort. The Illumina Infinium 450k Human DNA methylation Beadchip was used to obtain DNA methylation profiles across approximately 450,000 CpGs in cord blood samples. https://www.ncbi.nlm.nih.gov/geo/query/acc.cgi?acc=GSE69176.

An Epigenetic-Senescence-Signature, based on six CpGs, was either analyzed by pyrosequencing or by barcoded bisulfite amplicon sequencing. https://www.ncbi.nlm.nih.gov/geo/query/acc.cgi?acc=GSE82234.

### 2.3. Pyrosequencing

**Figure 1:**
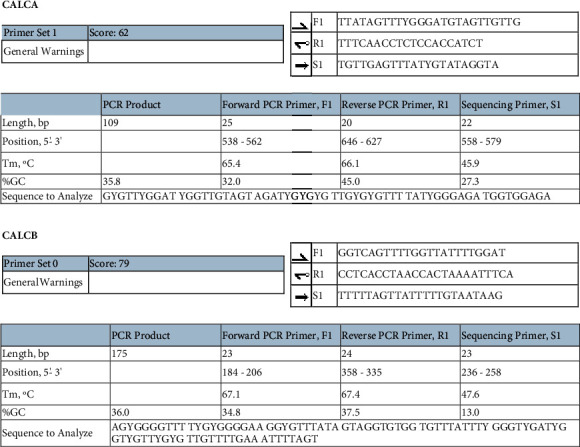
Primers, conditions, and sequencing sequences of CALCA and CALCB methylation.

DNA was extracted from umbilical cord tissues (about 5 cm from placenta attachment) of three infants with single chorionic triplets and preterm live birth after single blastocyst transplantation and double blastocyst transplantation double chorionic twins and two full-term live births using the Tiangen DNA Extraction Kit (TIANGEN, Beijing, China) according to manufacturer's instructions. DNA was sent directly to Gene Tech (Shanghai) Co, Ltd. (Gene Tech, Shanghai, China) for pyrosequencing to detect the methylation level of CpG island in CGRP promoter region. The sequencing primers, conditions, and sequencing sequences used are shown in Figure 1.

### 2.4. Immunofluorescence

The immunofluorescence measurements were performed as previously described [12]. The procedure is briefly described as follows: umbilical cord tissue and placental tissue were fixed in 4% formalin overnight, embedded in paraffin, sectioned at 4 mm. Immunofluorescence confocal microscopy was also undertaken to determine the correlation of CALCA (*α*CGRP) and CALCB (*β*CGRP). CALCA was detected with rabbit antihuman polyclonal antibody (1 : 50 dilution; A11804, Abclonal, CN), and CALCB was detected with rabbit antihuman polyclonal antibody (1 : 200 dilution; A5523, Abclonal, CN). The secondary antibody was rhodamine- (TRI-TC-) conjugated goat anti-rabbit IgG or FITC-labeled goat anti-rabbit IgG. Nuclei were stained with DAPI solution.

## 3. Results

### 3.1. Clinical Data of Identical Triple Pregnancies

A 29-year-old female patient was treated with in vitro fertilization (IVF) and transplanted a fresh cycle of one blastocyst (4AB) on day 5, which is a monochorionic triple amniotic pregnancy shown in pregnancy ultrasound. This blastocyst was premature rupture of membranes at 25 W + 1 week, and three boys were born prematurely. The newborns had an Apgar score of 4 and a weight of 800 g. One of the newborns was short in height and malnourished and died after rescue (Table 1). General clinical data show that the patient has lived with the current husband for 6 years, married for 5 years, and has an average sex life of 3-4/month. The husband has ejaculated and has not been pregnant yet. The patient was diagnosed with “polycystic ovary syndrome” in 2015. In 2016, the patient underwent a series of operations, such as laparoscopic ovarian perforation, hysteroscopy, and bilateral salpingectomy. Patient's infusion during surgery is normal. The pathological results after surgery showed endometrial polyps. Basic endocrine examination: FSH 1.11 IU/L, LH 6.4 IU/L, E2 35.2 miu/L, T 57.6 nmol/L, and AMH 5.95 ng/mL. Birth history: 0-0-0-0. Not pregnant for 6 years without contraception. The semen of the man is normal, the chromosomes of both men and women are normal, and the close relatives of both men and women do not have multiple births. All human studies were approved by the Ethics Committee of the First Affiliated Hospital of Fujian Medical University (MTCA, ECFAH of FMU [2019] 001) and (MTCA, ECFAH of FMU [2015] 084-1).

2019.8.23 Ultrasound showed 5 antral follicles on the left ovary, 27∗22 mm follicles on the right ovary, and 3-4 antral follicles, Em: 13.0 cm, C Type. GnRH-a ultralong regimen was used, and leuprolide was adjusted down 3.75 mg/d after communication with the patient. 2019.9.10 Leuprolide was used to downregulate for 27 days, blood hormone: E2: 33.19 pmol/l, FSH: 2.42 IU/L, LH: 0.51 IU/L, *P*: 0.64 nmol/L. Vaginal B-ultrasound shows that 8-9 antral follicles can be seen on the left ovary, 4 antral follicles can be seen on the right ovary, Em: 2.0 mm, C Type. After communicating with the patient, she was given LH 150 IU/day and HMG225 IU/d growth hormone 4 IU/d started. 2019-9-30 Gn11day, vaginal B-ultrasound: there are 22 follicles in both ovaries, of which 6 are >1.8 cm follicles. Blood hormone: E2: 7678 pmol/L, *P* 1.11 nmol/L, LH: 1.25 IU/L. Stop Gn, Azer 200ug trigger, OPU operation for 38 hours. 12 eggs were examined, 10 of which were mature. IVF short-term fertilization, seven appeared 2PN, and D3 high-quality 3 embryos. A fresh cycle embryo is transferred and administered: dydrogesterone 20 mg bid po∗10 d; progesterone suppository 2 tabletsbid pv∗10 d.

Frozen embryos: D5 (4AB, 4BB), D6 (4CC+).

**Figure 2:**
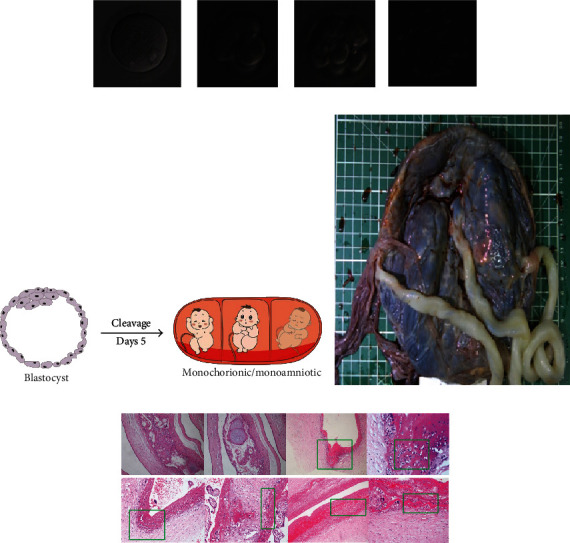
Clinical data of identical triple pregnancies. (a) Embryo development (2PN-4CII-8CII-4BA), scale 0.0002 mm; (b) identical triplet pattern diagram; (c) identical triple placenta umbilical cord and blood vessels on the umbilical cord (Left: general sample image of placenta and umbilical cord; upper right: pathological image of umbilical cord blood vessel of fetus A; middle right: pathological image of umbilical cord blood vessel of fetus B; lower right: pathological image of umbilical cord blood vessel of fetus); (d) chorioamnionitis and chorionic plate vasculitis, scale bar 0.003 mm; green box marketed and highlight show inflammatory cell infiltration area.

Fresh cycle embryo transfer: single blastocyst transplantation (2PN-4CII-8CII-4BA) (Figure 2(a)), the transplant process went smoothly. Day 11, blood HCG 194.7 mIu/mL, day 14, blood HCG 595.1 mIu/mL, 6 W + 4 ultrasound shows gestational sac size: 3.5∗1.4∗2.3 (cm). Gross pathology showed single placenta, three amniotic sacs, and three umbilical cords (Figures 2(b) and 2(c)). Histopathology showed acute chorioamnionitis and chorionic plate vasculitis (Figure 2(d)). No triplet transfusion syndrome was found.

### 3.2. Ultrasound Monitoring Results

**Figure 3:**
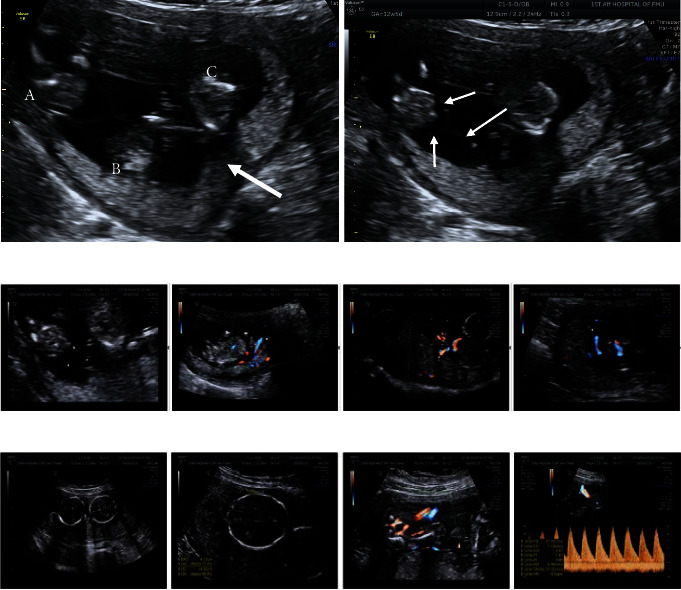
B-ultrasound monitoring results of identical triple pregnancies. (a) Left: three independent fetuses a, b, and c can be seen, and the arrow is a single placenta; right: three independent amniotic sacs are seen, and the arrow is the amniotic echo; (b) early color Doppler ultrasound NT examination; (c) ultrasonography in the second trimester (20 weeks).

Early ultrasound (12 W) of three independent fetuses (a, b, and c), a single placenta, and three independent amniotic sacs were shown in Figure 3(a). Color Doppler ultrasound NT examination revealed three fetuses and one placental echo in the uterine cavity, and the three fetuses were arranged in the upper right (a), upper left (b), and lower right (c) quadrants. Echoes of the diaphragm were seen among the three fetuses, and the three diaphragms and the placenta showed a T sign. The placenta was located in the posterior wall, covering the cervix by its lower edge. Maturity level was 0 (Figure 3(b)). Ultrasound examination in second trimester (20 weeks) revealed placental maturity level of I and thickness of about 3.23 cm (Figure 3(c)).

### 3.3. Database Search Results

The umbilical vein (6 samples) and cord blood (152 samples) were collected from the samples. The average methylation rate of CALCA was 10.08% and 9.50%, and that of CALCB were 5.31% and 7.71%, respectively.

### 3.4. Methylation Sequencing Results

The average values of CpG island methylation in CALCA promoter region in fetal A, B, and C umbilical cord tissues were 12.125%, 15.5%, and 18.625%, respectively, and these were higher than those in normal controls. The average methylation rate of CALCA promoter region in D and E umbilical cord tissues of full-term control fetuses was only 6.125% and 4.375%. Based on this, it was clear that the percentage of CALCA methylation in the umbilical cord tissues of premature fetuses was higher than that of full-term births, and the percentage of CALCA methylation in the C umbilical cord tissues of fetuses that died after rescue remained the highest.

**Figure 4:**
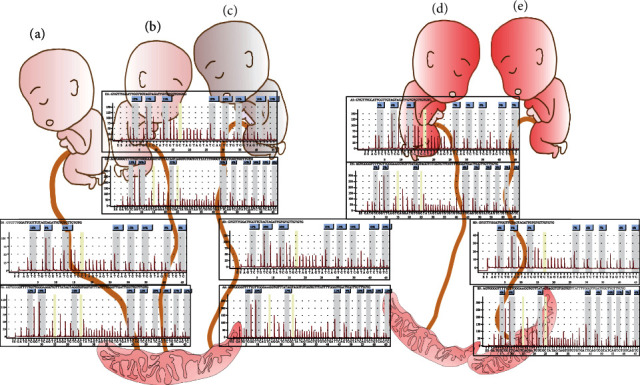
CALCA and CALCB methylation results of umbilical cord tissues from identical triple pregnancy and control. Among them, the identical triplets were triplet (a), triplet (b), and triplet (c) and triplet (d) and triplet (e) fraternal twins.

Similarly, the average values of CALCB methylation in umbilical cord tissues of premature fetuses A, B, and C were 15.889%, 11.222%, and 16.667%, respectively, and were higher than those of normal controls. However, the average percentage of methylation of CpG island in CALCB promoter region in D and E umbilical cord tissues of full-term fetuses was only 2.778% and 7.0%. In addition, the methylation percentage of CALCB in umbilical cord tissues of premature fetuses was higher than that of term births, and the CALCB promoter region methylation percentage in the C umbilical cord tissues of fetuses that died after rescue remained the highest (Figure 4).

Among these, the identical triplets were triplet A, triplet B, and triplet C and triplet D and triplet E fraternal twins. The results of pyrophosphorylation sequencing showed that the methylation levels of CALCA and CALCB in the umbilical cord tissue of three premature births with single chorionic sac were higher than those of full-term twins with double chorionic sac, and the methylation levels of death cases after rescue were higher when compared to others.

### 3.5. Tissue Immunohistochemical Test Results

**Figure 5:**
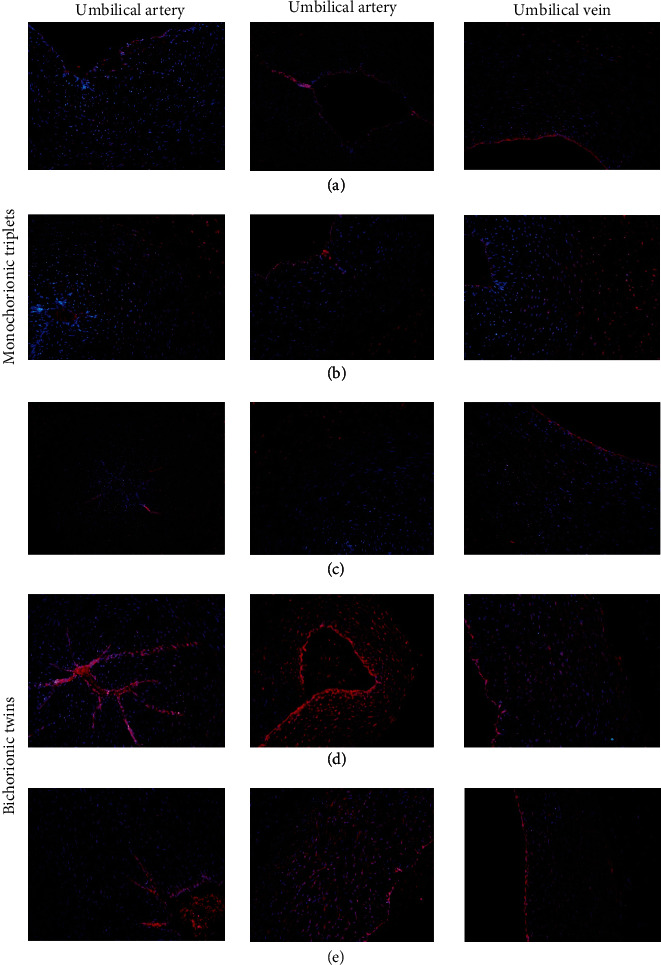
CALCA expression in the umbilical cord. Tissue section of umbilical cord were exposed to *α*CGRP IgG at 4°C for 20 minutes. The localization of *α*CGRP was monitored by immunofluorescence microscopy. In umbilical cord tissue, CGRP was weakly expressed, mainly in vascular endothelial cell layer. (a), (b), and (c) are identical triplets ((c) is the invalid death case); (d) and (e) are fraternal twins.

**Figure 6:**
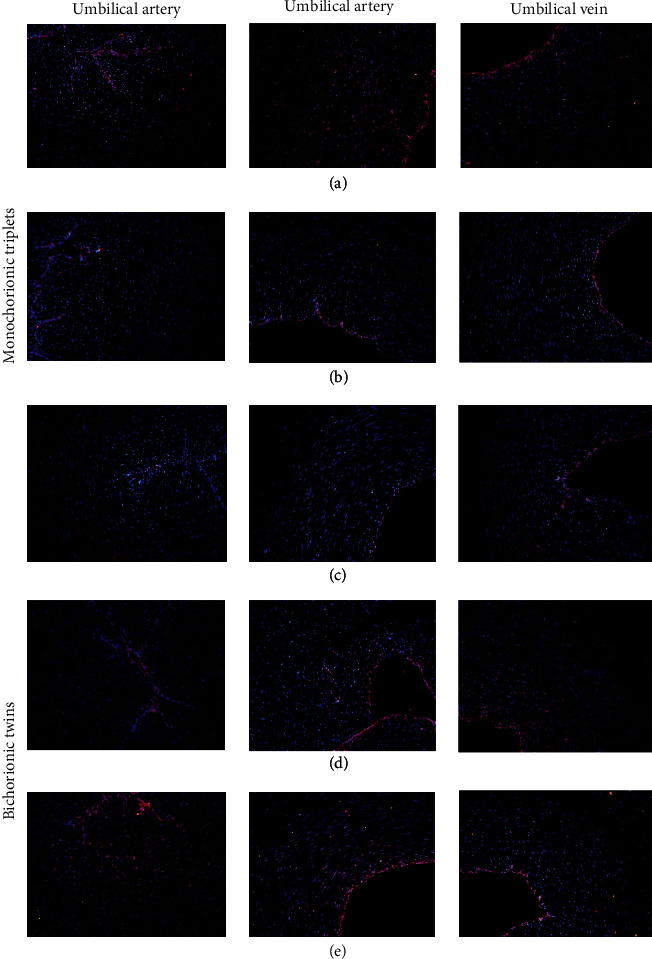
CALCB expression in the umbilical cord. Tissue section of umbilical cord were exposed to *β*CGRP IgG at 4°C for 20 minutes. The localization of *β*CGRP was monitored by immunofluorescence microscopy. In umbilical cord tissue, CGRP was weakly expressed, mainly in vascular endothelial cell layer. (a), (b), and (c) are identical triplets; (d) and (e) are fraternal twins.

Immunofluorescence results revealed that the expression of *α*CGRP in the umbilical cord of the twin brothers (triplet A, triplet B, and triplet C) was lower than that of normal controls (triplet D and triplet E), especially in death cases (triplet C) (Figure 5). Immunofluorescence results showed that the level of *β*CGRP revealed no significant differences in the vascular endothelium and vascular wall of umbilical cord of twin brothers and normal control, including the death cases without rescue (Figure 6).

## 4. Discussion

Premature infants are susceptible to many complications due to impaired immunity, inflammation, and vascular regulation [13, 14]. The discovery with regard to the relationship between calcitonin/calcitonin gene-related peptide (CT/CGRP) and pregnancy highlighted new signal transmission mediators in various physiological processes including reproduction [15, 16]. In addition to vascular regulation, CGRP comprises of a variety of functions in regulating immune and inflammatory responses [17–19]. It integrates inflammatory and immune responses in the local microenvironment [5, 6, 15]. CGRP can change lymphocyte proliferation, antigen presentation, cytokine production, B lymphocyte differentiation, and adhesion molecule expression [5, 6]. Studying the effects of neuropeptides on inflammation and vascular regulation can help us to uncover the possible mechanisms in preterm infants.

Our previous study has shown that mutations or abnormal splicing of CGRP gene could lead to abnormal localization and synthesis of CGRP in cells, causing perineuritis and vasculitis [5]. Similar to vascular endothelial cells and smooth muscle cells, trophoblast cells also expressed receptor proteins such as CRLR and RAMP1, suggesting that CGRP might be involved in the regulation of trophoblast function [20]. Long-term administration of CGRP receptor antagonist CGRP_8-37_ into pregnant rats significantly reduced the weight of young rats, increased systolic blood pressure and fetal mortality in a dose-dependent manner [21, 22]. This phenomenon suggests the relationship between the expression of CGRP and premature birth.

Preterm birth is associated with DNA methylation in several different tissues, including placenta, neonatal blood, and neonate-birth saliva [23, 24]. In these previous studies, many CpG loci associated with preterm birth were located in genes associated with neuronal development and/or neurodegenerative diseases [25, 26]. This might be one of the reasons for the differences in the development of identical fetuses [27, 28]. In this study, pyrosequencing proved that CALCA and CALCB methylation levels of preterm umbilical cord tissues of single-chorionic triple-amniotic sac were higher than those of double-chorionic twins and normal controls as derived from the database. Moreover, the methylation levels of death cases after rescue were higher when compared to the other two twin brothers. Immunofluorescence results showed that the expression of *α*CGRP was decreased in the blood vessel wall of the umbilical cord of monozygotic triplets, especially in death cases, but no significant difference was observed in *β*CGRP, whether it is compensatory or not requires further in-depth study.

CGRP hypermethylation might affect vascular adaptation of umbilical cord and function of trophoblast cells during pregnancy [29]. Therefore, we hypothesized whether high methylation of CpG island in the promoter region of CGRP gene in the umbilical cord tissues led to low expression of CGRP, promoting the development of preterm birth. The two subtypes of *α*CGRP and *β*CGRP have extremely high similarities and differ only in three amino acids, where one of them is lacking, and the other changes positively or negatively [30, 31]. Compared with the wild-type control, the *β*CGRP mRNA levels in the dorsal root ganglia and spinal cord of *α*CGRP knockout mice showed no change, but the levels in the intestine were reduced by two times [32]. In the intestines of mice lacking *β*CGRP, the expression of *α*CGRP messenger RNA was increased, indicating an adaptive mechanism to compensate the lack of *β*CGRP.

In conclusion, this study focused on premature live births of triple-chorionic tricotyledon after single-embryo transfer. The pyrosequencing method was used to detect the methylation of CALCA and CALCB in the umbilical cord and analyzed the normal control results of database search. This revealed that CALCA and CALCB hypermethylation might affect pregnancy vascular adaptation and trophoblast cell function by regulating the expression of CGRP. It is considered as an extremely important cause of premature birth and vasculitis.

## Figures and Tables

**Table 1:** General condition of newborns with three births with monochorionic three amniotic sac.

Fetus	Survival	Apgar score	Weight (g)	Height (cm)	Health status	Birth defects	Other
A	Yes	4	806	25	General	None	Healthy discharge
B	Yes	4	833	25	General	None	Healthy discharge
C	Yes	4	800	22	Unhealthy	None	Died after rescue

## Data Availability

All data and materials generated during and/or analysed during the current study are available from the corresponding author on reasonable request.
